# Behavioral Priming 2.0: Enter a Dynamical Systems Perspective

**DOI:** 10.3389/fpsyg.2017.01204

**Published:** 2017-07-18

**Authors:** Dario Krpan

**Affiliations:** Department of Social Policy, London School of Economics and Political Science London, United Kingdom

**Keywords:** dynamical systems, priming, behavior, entropy, complexity

## Abstract

On a daily basis, people are exposed to numerous stimuli, ranging from colors and smells to sounds and words, that could potentially activate different cognitive constructs and influence their actions. This type of influence on human behavior is referred to as priming. Roughly two decades ago, behavioral priming was hailed as one of the core forces that shape automatic behavior. However, failures to replicate some of the representative findings in this domain soon followed, which posed the following question: “How robust are behavioral priming effects, and to what extent are they actually important in shaping people's actions?” To shed a new light on this question, I revisit behavioral priming through the prism of a dynamical systems perspective (DSP). The DSP is a scientific paradigm that has been developed through a combined effort of many different academic disciplines, ranging from mathematics and physics to biology, economics, psychology, etc., and it deals with behavior of simple and complex systems over time. In the present paper, I use conceptual and methodological tools stemming from the DSP to propose circumstances under which behavioral priming effects are likely to occur. More precisely, I outline three possible types of the influence of priming on human behavior, to which I refer as emergence, readjustment, and attractor switch, and propose experimental designs to examine them. Finally, I discuss relevant implications for behavioral priming effects and their replications.

## Introduction

Behavioral priming has recently been subjected to harsh criticisms, primarily because of numerous replication failures (e.g., Doyen et al., 2012; Pashler et al., 2012; Harris et al., 2013; Shanks et al., 2013; Rohrer et al., 2015; Wagenmakers et al., 2015). This has resulted in various recommendations regarding how to improve research practices, largely focused on conducting high-powered studies, pre-registration, and replication (e.g., Francis, 2012; Wagenmakers et al., 2012; Asendorpf et al., 2013; Cesario, 2014; Stroebe and Strack, 2014; Jonas and Cesario, 2015; Simonsohn, 2015). The question is, however, whether behavioral priming has been such a controversial topic because of undesirable research practices (e.g., underpowered studies), or because researchers have been using inappropriate methodological tools to investigate priming effects. In the present article, I argue that, for the “crisis” of priming to be resolved, this phenomenon needs to be tackled from a dynamical systems perspective (DSP; see Vallacher and Nowak, 1994, 1997; Nicolis and Prigogine, 1977; Beer, 2000; Guastello et al., 2009; Wiese et al., 2010). More specifically, by relying on the DSP, I identify precise circumstances under which priming can influence behavior and present appropriate methodological tools for modeling the behavioral effects. Finally, I discuss how failing to consider the DSP when designing behavioral priming research can lead to failures in detecting and replicating the effects.

## What is behavioral priming?

Behavioral priming refers to the notion that exposing people to an external stimulus (e.g., a list of words describing old people) activates a mental construct associated with this stimulus (e.g., “being old”), which may in turn affect overt behavior without the actor necessarily being aware of this influence (e.g., Bargh et al., 1996). Whereas most researchers agree that priming operates by making a mental construct accessible (e.g., Loersch and Payne, 2011; Schröder and Thagard, 2013; Fujita and Trope, 2014; Klatzky and Creswell, 2014; Stroebe and Strack, 2014; Wentura and Rothermund, 2014; Barsalou, 2016), it remains debatable how and when exactly this accessibility should influence behavior. There are generally two different types of theories regarding the prime-behavior link (see Fujita and Trope, 2014). More traditional approaches (e.g., Dijksterhuis and Bargh, 2001) suggest that enhanced construct accessibility (e.g., “being old”) automatically activates the tendency to execute associated behaviors (e.g., walking slowly). Therefore, these approaches are based on the ideomotor principle (James, 1890), according to which thinking of a behavior automatically evokes processes necessary to execute it. In contrast, recent theoretical models (e.g., Loersch and Payne, 2011; Klatzky and Creswell, 2014; Barsalou, 2016) emphasize “more active interpretation and meaning-making processes as key determinants of priming phenomena” (Fujita and Trope, 2014, p. 72). Broadly speaking, these models suggest that a mental construct activated by priming is just one of the inputs the decision maker needs to consider, alongside one's goals and various situational factors, when determining how to best respond to the situation at hand. The present article does not intend to criticize the existing models or theorize on specific mechanisms through which primes impact behavior. Instead, it uses conceptual and methodological tools stemming from the DSP to provide a unique outlook on basic psychological forces that constrain priming effects and to examine how to effectively model these effects. To accomplish this objective, I next introduce the relevant DSP constructs.

## Fundamentals of the dynamical systems perspective

Grasping the DSP requires unveiling the meaning behind “dynamical” and “systems.” Different scientific disciplines and streams tend to define the notion of a system in different ways. From a viewpoint of certain theorists who work within the realm of the DSP, a system is broadly defined as “a theoretical construct that simplifies nature” (Ward, 2002, p. 46) and involves precise mathematical modeling (see also Beer, 2000). Indeed, many real-world phenomena, ranging from the human brain to an ecosystem, are highly complex and consist of numerous interconnected elements that operate on many different scales and whose interactions constitute the phenomena (Vallacher et al., 2002b, 2015; Ward, 2002; Stephen et al., 2009b; Wiese et al., 2010). For example, in the context of behavioral priming, a behavior (e.g., eating, walking, solving intellectual problems) involves interactions among different bodily organs, cells constituting these organs, chemical elements constituting the cells, atoms constituting the chemical elements, and different subatomic particles constituting the atoms. All these elements and their interactions across and within the scales on which they operate would be highly difficult to model individually, at least with the technologies that are currently available, and hence, from the dynamical systems perspective (DSP), human behavior can be more effectively studied if it is conceptualized as a system. To conceptualize a phenomenon such as human behavior as a dynamical system means to model its change over time by focusing on a smaller number of variables that capture its essence. For example, a simple way to model eating as a dynamical system would involve investigating how the quantity of food consumed changes per intervals (e.g., 1 min) of a certain period (e.g., 20 min). Alternatively, one could involve additional variables such as arousal and model their role in eating over time. I will get deeper into mathematical representations of dynamical systems as I introduce other relevant DSP constructs.

So far, the DSP has been implemented in numerous academic disciplines, ranging from mathematics and physics to biology, neuroscience, economics, and psychology (Gleick, 1987; Vallacher and Nowak, 1997; Gregson and Guastello, 2011). This indicates that the paradigm has been highly effective in studying various natural phenomena, but also that its tools and concepts have been shaped by each of these disciplines, thus leading to certain terminological inconsistencies. For example, in relation to dynamical systems, many different theoretical labels have been used, such as dynamical systems theory (Vallacher and Nowak, 1994, 1997; Nicolescu and Petrescu, 2013), dynamical systems approach (Finkenstädt and Grenfell, 2000), DSP (Beer, 1995; Shenoy et al., 2013), non-linear dynamics (Stephen et al., 2009b), complex dynamical systems (Richardson et al., 2014), etc. In the present paper, I use the DSP as a broad term that comprises dynamical systems theory (DST), which evolved to tackle simple systems that can be modeled with high accuracy using only few variables (e.g., mathematical pendulum; Robinson, 1995; Gros, 2008), and complex systems theory (CST), which provides more advanced tools to study phenomena of higher degrees of complexity (e.g., ecosystems, living cells, human cognition, and behavior, Prigogine and Stengers, 1984; Gilden et al., 1995; Ward, 2002; Gros, 2008). In psychology and biological sciences, which usually deal with complex systems, these two theories are often conflated for a good reason: DST provides basic conceptual and computational tools that, despite being aimed at simple deterministic systems, can also be applied to more complex systems and are a prerequisite for understanding them—CST is therefore an extension of DST (see Gros, 2008; Guastello and Gregson, 2011; Butner et al., 2015). In the present paper, I hence treat DST and CST as a continuum under the umbrella term of the DSP, but it is important for readers to understand terminological intricacies to delve deeper into dynamical systems literature.

**Figure 1:**
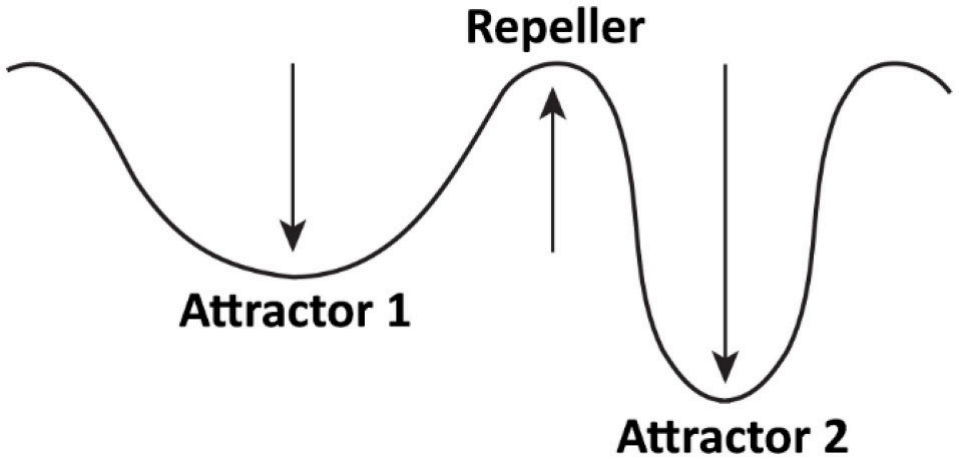
A conceptual attractor model of restrained eaters' eating behavior. Attractor 1 corresponds to restrained eating, whereas Attractor 2 corresponds to enhanced eating. Repeller corresponds to an eating pattern that falls between enhanced and restrained eating and is unlikely to occur.

One of the most important concepts stemming from the DSP is that of an attractor—a stable state toward which a system evolves over time. Attractors can be illustrated in two different ways—conceptually and mathematically. I will start with a conceptual explanation. Figure 1 depicts a hypothetical attractor model of restrained eating. Based on previous research (e.g., Heatherton et al., 1991; Fedoroff et al., 1997; Harris et al., 2009), it is plausible that restrained eaters have two attractors for eating: one corresponding to restrained eating (Attractor 1), and one to enhanced eating (Attractor 2). The valley representing each attractor—formally known as the basin of attraction—denotes the strength of an attractor (depth) as well as its likelihood of occurrence (width). Attractor 1 has a wide basin, which indicates that restrained eaters are on average likely to restrain their food consumption (Rideout and Barr, 2009). However, the basin is also shallow. Therefore, certain situational circumstances such as priming (Fedoroff et al., 1997) or anxiety (Heatherton et al., 1991) can influence the eating behavior to leave Attractor 1 and settle in Attractor 2. This attractor has a deep basin of attraction and is therefore strong, which means that, once activated, it may be difficult for restrained eaters to stop overeating. The hill between the two attractors represents a repeller—an unstable state unlikely to occur (Butner et al., 2015); for example, eating that is neither restrained nor enhanced.

The above-reviewed attractors are referred to as fixed point, or set point attractors (Butner et al., 2015; Vallacher et al., 2015). These are, however, not the only attractor types. Other possible attractors include limit cycles, strange attractors, or chaotic attractors (for an overview, see Barton, 1994). In the present paper, the focus is on fixed point attractors because I find them relevant in relation to priming, whereas other attractors may not be as useful given the type and quality of data that priming research can produce. In the context of fixed point attractors, it is important to know that a behavioral system does not always need to be characterized by two attractors, as in the example of restrained eating. In fact, some behavioral systems may have more than two attractors, and some only one. For example, behavior of unrestrained eaters can be characterized by a single attractor, given that their eating does not considerably change in different circumstances (e.g., Fedoroff et al., 1997).

Let us now move beyond conceptual explanations and examine mathematical formulations of fixed point attractors. The primary mathematical tool for modeling dynamical systems constitutes differential equations which describe how certain variables comprising a system change over time (Beer, 2000; Gros, 2008). As an example, I will start with a simple first order differential equation (see Butner et al., 2015):

(1) $$dx/dt_{i}=b_{0}+b_{1}x_{i}$$

In this equation, the expression *dx/dt*_*i*_ (it can also be written as ẋ) corresponds to a change in variable *x* (to continue the previous example, we can assume that *x* represents the quantity of food consumption in grams) per certain temporal interval (for example, 1 min). Furthermore, *b*_0_ is a constant, whereas *b*_1_ corresponds to the slope of change. In a nutshell, the equation specifies that the change in *x* per temporal increment *dt* follows a linear pattern.

**Figure 2:**
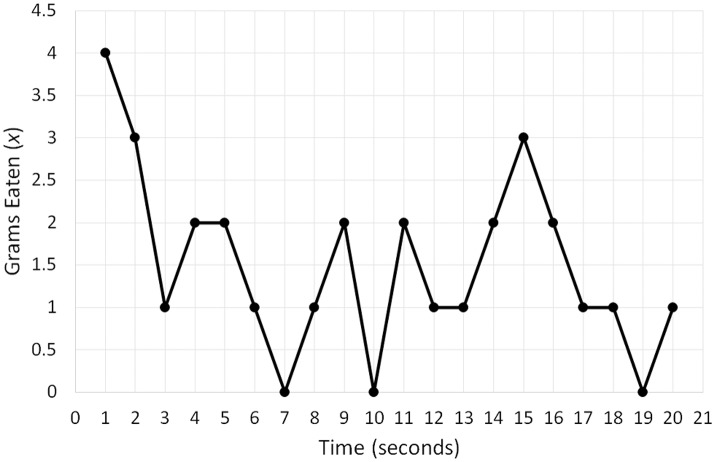
A hypothetical time series of the quantity of snacks in grams (x) a person consumed per 1-min intervals of a 20-min period.

To bring this equation to life, I will use a hypothetical dataset comprising a person's eating pattern over a 20-min period. Figure 2 depicts a time series of the quantity of snacks in grams (*x*) the person consumed per 1-min intervals of this period. We can see that the person started by eating four grams during the first minute, which then dropped to three grams, and during the final minute the consumption was one gram. To implement Equation (1), we need to transform the time series into a variable that corresponds to the change in food consumption: *dx*/*dt*. This variable is obtained by calculating the difference between adjacent data points in the series. For example, for the first five points (4, 3, 1, 2, 2) the difference is −1, −2, 1, and 0 (Butner et al., 2015; Wong et al., 2016). To obtain specific parameters for Equation 1, we need to fit the equation to variables *dx*/*dt* and *x*. Given that the formula essentially represents linear regression, the fitting is done by conducting a regression analysis, with *dx*/*dt* as a dependent variable and *x* as a predictor (the final data point needs to be removed from *x* for the two variables to have equal length).

**Figure 3:**
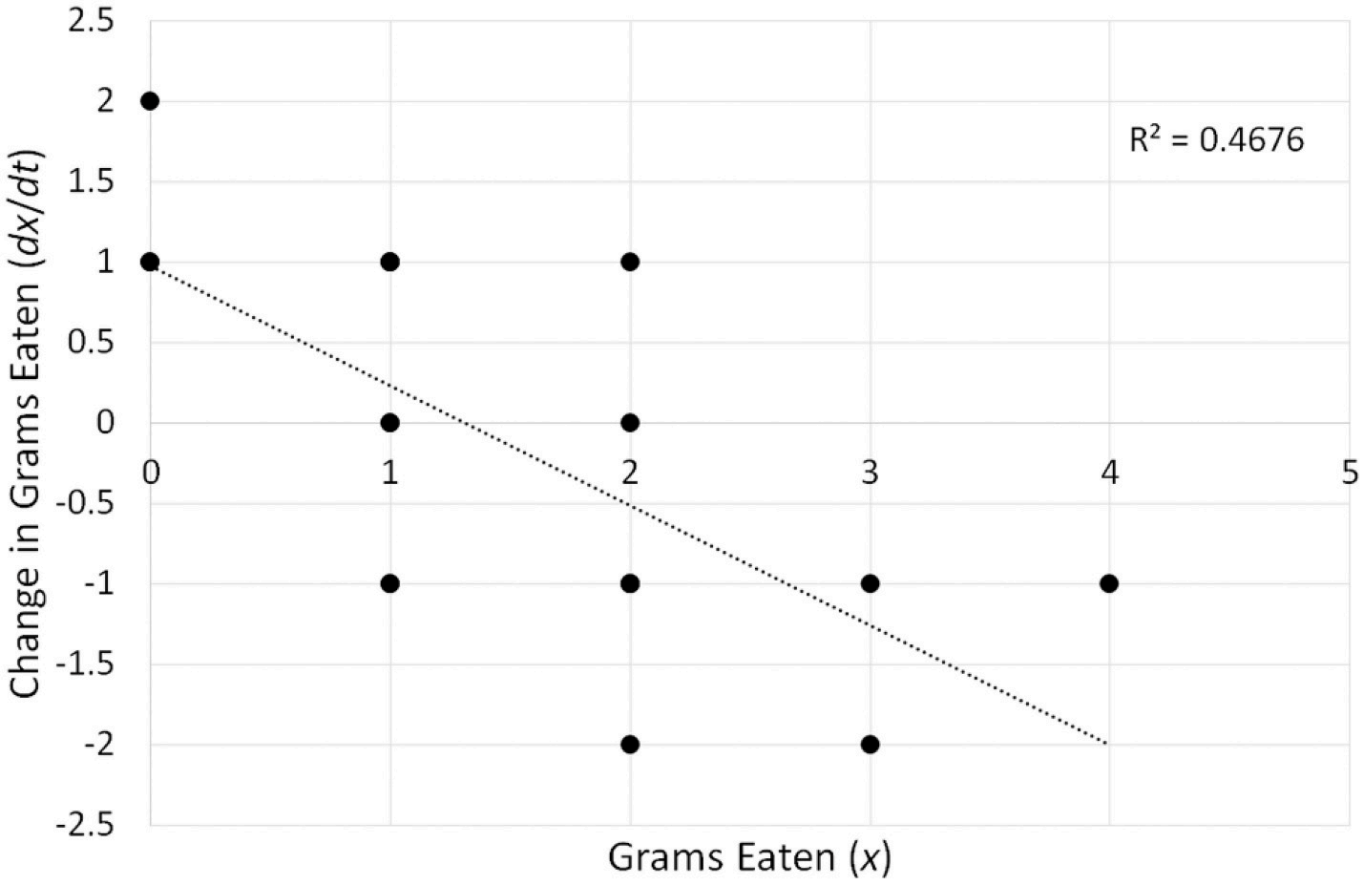
A graphical representation (topology) of a differential equation (*dx*/*dt* = 0.977−0.744*x*) reflecting the change in food consumption over a 20-min period depicted in Figure 2. The point at which the best-fit line crosses the x-axis (1.313) corresponds to the fixed-point attractor—the stable value of food consumption toward which the eating pattern evolves over time.

Fitting the model (*R*^2^ linear = 0.4676) produces the following best fit line: *dx*/*dt* = 0.977−0.744*x*. Figure 3 constitutes a graphical representation of this model. In “dynamical” terminology, the graphical representation of differential equations is known as topology, and different names such as state space or phase space are also used (e.g., Spivey and Dale, 2006; Shelhamer, 2011; Butner et al., 2015). The point where the best fit line crosses the *x*-axis corresponds to the fixed point. If the slope is negative, the fixed point is an attractor, and if the slope is positive, it is a repeller (see Butner et al., 2015). In our example, we have therefore identified an attractor, and to calculate its value, we need to set *dx*/*dt* to 0, which indicates the point of no change, and solve the equation 0 = 0.977−0.744*x*. The solution corresponds to 1.313, which is the attractor. The strength of the attractor is conveyed by the magnitude of the slope of the best fit line (*b*_1_ = −0.744 in our example). The larger the magnitude, the stronger an attractor, thus indicating that the system quickly moves toward the fixed point (Butner et al., 2015).

Equation (1) is relatively simple and allows identifying only one fixed point attractor, which means it can be used to model behaviors such as eating of non-restrained eaters or even of restrained eaters in situations where only one attractor would be expected (e.g., only restrained eating). However, to identify more attractors in a time series, a more elaborate equation would be needed. The most effective way to proceed in this respect would be fitting polynomial regression models of different order (Butner et al., 2015; Wong et al., 2016). Linear regression itself corresponds to a polynomial regression model of a first order (Equation 1) and can capture only one fixed point; a quadratic expression corresponds to a second order polynomial regression model (Equation 2) and can identify two fixed points; a cubic expression corresponds to a third order polynomial regression model (Equation 3) and can capture three fixed points, and so on (Draper and Smith, 1998).

(2) $$dx/dt_{i}=b_{0}+b_{1}x_{i}+b_{2}x_{i}^{2}$$

(3) $$dx/dt_{i}=b_{0}+b_{1}x_{i}+b_{2}x_{i}^{2}+b_{3}x_{i}^{3}$$

Therefore, based on predetermined theoretical predictions, one can fit different polynomial regression models to time series data and compute fixed point attractors or repellers using a similar procedure as described in the previous paragraph (for more details, see Mourrain and Pavone, 2009; Butner et al., 2015).

Beyond fitting more complex differential equations, the intricacy of dynamical systems modeling increases if multiple dimensions are considered. A dimension of a dynamical system corresponds to the number of mathematical variables used to describe its behavior (Shelhamer, 2011; Butner et al., 2015). In the previous examples, we tackled only one variable (*x*), and the equations we employed expressed how this variable changes over time. In other words, we were modeling a one-dimensional system. However, perhaps we may want to investigate how two variables change simultaneously over time; for example, how a person's mood changes alongside eating, and whether they exhibit certain stable states. In that case, we would need to measure both the amount of food eaten and the person's mood per 1-min increments. This dynamical system would need to be modeled using two differential equations rather than one (in general, one dimension corresponds to one differential equation, and multidimensional systems consist of multiple differential equations) and solve them simultaneously to calculate attractors, repellers, and their strength. For a tutorial on how to tackle multidimensional dynamical systems and their topologies, see Butner et al. (2015).

Although one can model natural systems by fitting various differential equations, there are certain limitations in this regard. First, the more complex a system gets, the less easy it is to identify the appropriate equations and dimensionality, in which case one may need to make a pragmatic decision to fit relatively oversimplified models that do not involve all the important variables capturing the system's essence (Gros, 2008). In this case, certain complexity measures can yield deeper insights into the system's intricacies. Second, to model some of the most important properties of natural systems that are highly relevant to priming—self-organization and emergence (these will be tackled in Section Emergence)—it is necessary to capture how a system's complexity changes over time (Stephen and Dixon, 2009; Stephen et al., 2009a,b; Dixon et al., 2010).

The number of measures that quantify complexity is immense (Pincus, 1991; Wackerbauer et al., 1994; Bar-Yam, 1997; Rezek and Roberts, 1998; Lloyd, 2001; Marwan et al., 2002; Wagenmakers et al., 2004; Webber and Zbilut, 2005; Gros, 2008; Gershenson and Fernández, 2012). A family of complexity measures of highest relevance within the present article is the entropy family (Guastello, 2011c). Entropy was first defined in the realms of thermodynamics and statistical mechanics (Carnot, 1824; Clausius, 1865; Boltzmann, 1886/1974; Atkins, 1984; Ben-Naim, 2007; Lemons, 2013), where it captures the complexity of the microscopic structure of different thermodynamic systems. A more relevant entropy measure in the present context is information entropy (Shannon, 1948), given that it conveys the complexity of a string of symbols (e.g., letters or numbers), such as a time series. The formula for information entropy is as follows:

(4) $$H=-\sum_{i}^{n}p(x_{i})log_{2}p(x_{i})$$

This formula computes the quantity of information contained in a string (in *bits*) by capturing the probability of occurrence of each symbol within the string.

**Figure 4:**
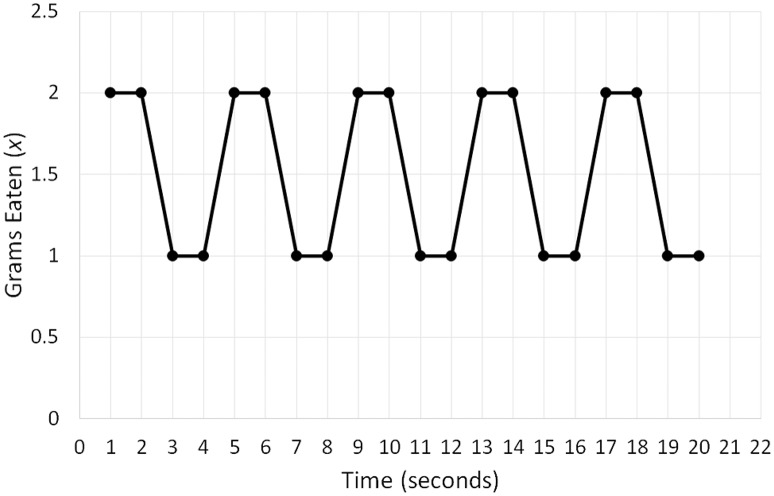
A hypothetical time series of the quantity of snacks in grams (x) a person consumed per 1-min intervals of a 20-min period characterized by low information entropy.

**Figure 5:**
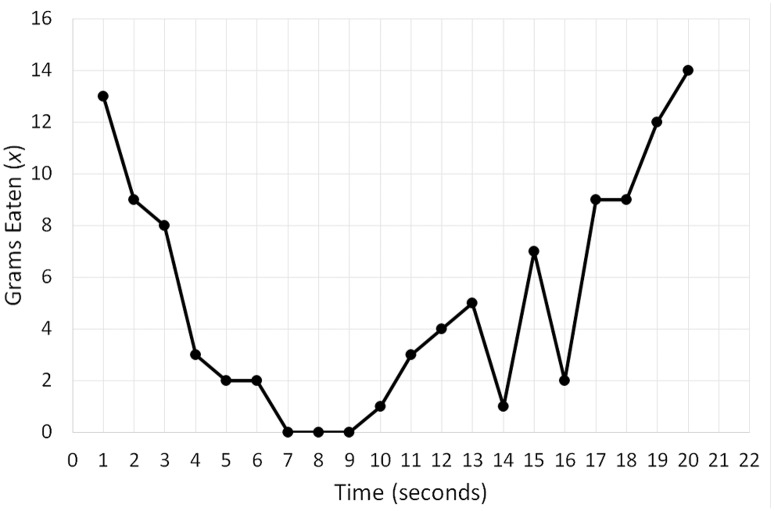
A hypothetical time series of the quantity of snacks in grams (x) a person consumed per 1-min intervals of a 20-min period characterized by high information entropy.

To clarify Equation (4), I will use two hypothetical time series (Figures 4, 5) quantifying the snacks (*x*) a person consumed per 1-min intervals of a 20-min period. The distribution of variable *x* in Figure 4 can be summarized as String 1 (2, 2, 1, 1, 2, 2, 1, 1, 2, 2, 1, 1, 2, 2, 1, 1, 2, 2, 1, 1), whereas its distribution in Figure 5 can be summarized as String 2 (13, 9, 8, 3, 2, 2, 0, 0, 0, 1, 3, 4, 5, 1, 7, 2, 9, 9, 12, 14). Even intuitively, we can already see that String 2 is more complex than String 1 because it contains a larger variety of symbols (= different numbers). Computing information entropy for each string indeed confirms this assumption: *H* (String 1) = 1 bit; *H* (String 2) = 3.41 bits. If we attempted to compute the attractors in the two datasets by fitting different polynomial regression models, we would see that the first time-series has only one fixed point attractor because it is possible to fit only the linear regression model. However, the second time-series yields a better fit with higher-order polynomial regression models (e.g., order 4) and has more than one fixed point. Thus, as entropy of a system increases, more complex attractor structures that may be difficult to capture become possible, and clear attractors may even be absent.

Overall, the present section introduced some of the basic DSP concepts and mathematical tools I will later extend and apply to behavioral priming. However, the DSP comprises an immense body of concepts and mathematical tools, and readers who want to get a deeper understanding of this paradigm can consult resources from the **Appendix**. In the next section, I examine the link between the DSP and behavioral priming to specify the circumstances in which this phenomenon is likely to occur and propose how to test it.

## Connecting the dynamical systems perspective to behavioral priming

Although tools and concepts stemming from the DSP have been introduced to psychology (Barton, 1994; Vallacher and Nowak, 1994, 1997, 2007; Ayers, 1997; Carver and Scheier, 1998; Guastello et al., 2009), only three studies that classify as priming have adopted its insights so far (Wegner et al., 1984, 1986; Vallacher et al., 2002a). Moreover, they were conducted before major advancements in psychological applications of the DSP methodology took place (Riley and Van Orden, 2005; Guastello and Gregson, 2011; Holden et al., 2013; Butner et al., 2015). To show that, despite being overlooked by priming researchers so far (for potential reasons, see Gelfand and Engelhart, 2012), the DSP can provide crucial insights into behavioral priming, I will first consider this phenomenon through the prism of “dynamical” terminology.

In this context, priming can be defined as an external perturbation of a behavioral system (Vallacher et al., 2002a, 2015). External perturbations are any influences that act on a dynamical system and do not arise from the elements that constitute the system but from the surroundings (Prigogine and Stengers, 1984; a system can also be internally perturbed, see Bender and Orszag, 2013). For example, a system of two people talking to each other can be perturbed by loud noise from the surroundings, whereas the workings of the human body can be perturbed by a sudden temperature change. Based on previous theorizing on priming, it is plausible that primes perturb a behavioral system by evoking cognitive constructs that have automatic associations with the motor system and/or by serving as inputs into decision-making processes that shape behavior (e.g., Dijksterhuis and Bargh, 2001; Loersch and Payne, 2011; Klatzky and Creswell, 2014).

A remarkable characteristic of many natural systems is that their behavior can resist even strong external perturbations and maintain stable attractor dynamics (Prigogine and Stengers, 1984). An obvious example is human behavior. We are exposed to numerous stimuli at any given moment, ranging from different sounds and objects to other people with whom we interact, and yet, considering all these perturbations, our behavior seems relatively constant. Indeed, we can maintain purposeful and goal directed behaviors and are not completely at the mercy of occurrences in our environment (Austin and Vancouver, 1996). In fact, some behaviors, such as addictions or habits, are so difficult to alter that major perturbations, such as interventions from friends and family members, are necessary to change them (Aarts and Dijksterhuis, 2000; Duhigg, 2012).

Considering all events a person may daily encounter, primes are relatively minor perturbations. Indeed, priming researchers posit that primes are subtle cues that usually impact behavior outside of awareness (Bargh, 2006). Hence, if one considers the DSP, primes may be construed as control parameters that could alter behavioral dynamics only in very specific circumstances (see Kelso, 1995), and it is unlikely that they generally shape people's actions in a more robust manner. In line with this assumption, I propose there are three different types of influence to which I refer as (a) Emergence; (b) Readjustment; and (c) Attractor switch. In the next sections, I provide the rationale behind these influences, discuss when they should occur, and propose how to investigate them.

### Emergence

Situations in which priming may be most likely to impact behavior are the ones where clear cognitive patterns that shape action have not yet emerged, and the behavioral system therefore lacks stable attractors (Vallacher et al., 2015). In fact, cognitive psychologists have used the concept of entropy to describe such a complex mental state (Hirsh et al., 2012). To think of the link between cognition and entropy, let us conceptualize cognition as a string of symbols corresponding to different possible perceptions or interpretations of a situation. In a cognitive state called String 1 (1, 1, 1, 1, 2, 1, 1, 1, 1, 1, 1, 2), there is one dominant interpretation of a situation, denoted by number 1, and if we apply Equation (4) to this string, the calculated entropy will be low (*H* = 0.65 bits). In contrast, in a cognitive state called String 2 (1, 2, 3, 4, 5, 6, 7, 8, 9, 10, 11, 12), there are many different interpretations, and the entropy is higher (*H* = 3.58 bits). String 1 therefore corresponds to a coherent cognitive state that may be associated with clear action patterns, whereas String 2 corresponds to an incoherent, “entropic” state possibly linked to ambiguous action patterns.

In literature stemming from the DSP, systems in high-entropy states are labeled as “far-from-equilibrium” and tend to *self-organize* (Gershenson and Heylighen, 2003; Guastello and Liebovitch, 2009). Self-organization is one of the main qualities of complex systems in nature (Prigogine and Stengers, 1984). By continuously interacting over time, elements of a system in a state of high entropy can organize themselves into stable structures, thus reducing the entropy. The process is called self-organization because it is not controlled by some central supervisory unit and occurs through continuous interactions among the elements. Highly entropic systems are characterized by inefficient use of energy; therefore, whenever a system's entropy level reaches a critical point, the system needs to self-organize into a new stable state (Guastello and Liebovitch, 2009). The transition of a system from a disordered into ordered state is known as *emergence* (Stephen et al., 2009b).

Whereas self-organization and emergence have primarily been construed in relation to thermodynamic entropy (Prigogine and Stengers, 1984), recent advancements in cognitive psychology showed that information entropy contributes significantly to the scientific understanding of self-organization in human cognition (Dixon et al., 2010). For example, a hypothetical cognitive state called String 2 that I introduced earlier has high information entropy and may therefore self-organize into the stable state represented by String 1. This way, the cognitive system would maintain its efficiency and remain functional in meeting situational demands.

Beyond theoretical speculations, how is cognitive entropy actually measured? This cannot be done via self-reports because people do not have the required level of insight (Nisbett and Wilson, 1977) and it would be impossible to capture short-term cognitive changes necessary to compute cognitive entropy by relying on this method. Therefore, based on the principle of embodied cognition that human mind is grounded in the body (Glenberg and Kaschak, 2002; Wilson, 2002), cognitive psychologists found a way to access cognitive dynamics by measuring the dynamics of bodily movements (Vallacher et al., 1994, 2015; Magnuson, 2005; Spivey et al., 2005; Spivey and Dale, 2006; Spivey, 2007; McKinstry et al., 2008; Duran et al., 2010, 2013; Freeman and Ambady, 2010; D'Mello et al., 2012; Pärnamets et al., 2015; Wong et al., 2016).

To clarify this, I will rely on research by Stephen et al. (2009b). They investigated cognitive dynamics involved in solving gear-system problems. A typical problem consists of a series of interconnected gears; the task is to predict the movement of the last gear from the movement of the first one. To capture changes in participants' cognitive entropy during problem solving, the authors tracked their hand motion and computed information entropy from a time series of angular velocity of this motion over time. In the initial problem solving stage, participants would typically follow each gear in the sequence to predict the movement of the final gear. This stage was marked by highly variable hand motion, thus indicating high cognitive entropy. Then, something extraordinary happened: after cognitive entropy reached its peak, participants started realizing that all even (odd) gears in the sequence turn in the same direction, and the movement of the final gear can be determined by counting whether it is even/odd. This insight was marked by entropy decrease. Thus, an initially entropic cognitive state (not having a clear view of how to best solve gear-system problems) emerged into a new lower entropy state (knowing how to effectively solve the problems). This cognitive change was related to a behavioral change—emergence of a new behavioral attractor—given that the time series of the number of gear problems solved over time was remarkably different after (vs. before) participants realized the efficient problem-solving rule.

Considering the process of emergence, why would behavioral priming effects be most potent under high cognitive entropy? When cognition is in an unstable, far-from-equilibrium state, its sensitivity to external perturbations increases because it uses them as information that will guide the emergence of a new stable state (Vallacher et al., 2015). In that case, even seemingly irrelevant information such as primes may be incorporated by the brain into cognitive structures that shape behavior. Although this assumption has not yet been demonstrated by employing motion tracking and modern dynamical methods, it was supported by Wegner et al. (1984, 1986) in the context of Vallacher and Wegner (1987, 1989, 2014) action identification theory.

According to the theory, people construe their actions in low or high level terms. For example, locking a door can be thought of as putting a key in the lock (low-level interpretation) or securing the house (high-level interpretation). Because low-level interpretations are detail-oriented and lack meaning and purpose, they may reflect high-entropy cognitive states, whereas high-level interpretations provide meaning behind actions and may thus reflect low-entropy states. Wegner et al. (1984, 1986) indeed showed that, under low-level understanding of a situation, contextual information such as primes impact behavioral intentions and lead to emergence of a high-level understanding. For example, when participants interpreted an experiment they just completed in low (vs. high) level terms, they were more likely to comply with a random statement proposing a high-level reason behind their participation (e.g., an altruistic reason to help the experimenter), thus suggesting that the high-level interpretation emerged from arbitrary external information (Wegner et al., 1986). Furthermore, the statement primed them to indicate they would be more likely to participate in a future activity that is motivationally congruent with the concept evoked by the statement (e.g., altruism). Although the data collected by Wegner et al. (1986) are not suitable for precise dynamical modeling that would allow computing attractors as in Butner et al. (2015), one could specify attractors conceptually. Being more “attracted” to undertake a prime-congruent activity (A) rather than another activity (B) indicates that activity A itself is the attractor. For other research linking priming to high-entropy situations, see Keefer et al. (2011) or Mussweiler and Strack (2000).

Overall, in this section I argued that priming should influence behavior in the context of emergence. In the next section, I further decompose this assumption using precise methodological language and propose how to test it.

#### Investigating emergence

Emergence is a multilayered process; hence, investigating behavioral priming within its realm is not an easy task. I suggest four crucial components need to be considered when devising an appropriate research design: priming, situation, cognition, and behavior. Behavior is the core component and needs to be carefully selected, given that it will determine how other elements are tackled and how the data are analyzed. Broadly speaking, behaviors can be classified into two types—those that are time-series compatible, and those that are not. Some behaviors, such as eating (Harris et al., 2009), solving intellectual tasks (Dijksterhuis and Van Knippenberg, 1998), or walking (Bargh et al., 1996; Cesario et al., 2006) can be expressed as time series, assuming they are assessed over an appropriate duration. In this regard, the main measure of interest is usually a specific quantity conveying “how much of a behavior” has been accomplished (e.g., grams of foods eaten, distance traveled, number of tasks correctly solved, etc.), and this quantity can be assessed per specific time intervals (e.g., every 30 s, every minute). However, some behaviors are time-series incompatible; for example, one-off decisions that constitute single acts, such as buying wine (North et al., 1997), choosing a product (Chartrand et al., 2008), or voting (Hassin et al., 2007). These behaviors could in principle be repeated numerous times and therefore expressed as time series. However, this would create different kinds of issues that may attenuate the interpretability of the data. For example, a researcher may record the shopping behavior of a person purchasing groceries online or in a supermarket and compute the number of products selected or the amount of money spent per time unit. However, these products may greatly differ in terms of packaging, price, health value, etc., and it would therefore be more relevant to focus on the types of products in the shopping cart rather than on the quantity purchased per certain temporal interval. Overall, time-series incompatibility reflects the general observation that some standalone actions may not be suitable for time-series analysis.

To investigate priming under emergence, a behavioral time series should ideally be used because this allows computing attractors as described in Section Fundamentals of the Dynamical Systems Perspective (Butner et al., 2015). Otherwise, the researcher would need to treat attractors metaphorically and assume that choosing action A (over B) indicates that A itself is the attractor. In the context of time-series compatible behaviors, an important decision to make is how many data points one should measure. A general rule is—the more data points the better. Emergence is a subtle process, and having too few data points may decrease the sensitivity with which important behavioral changes throughout this process can be captured. An accurate rule on this issue has not been clearly specified. Although required time samples will vary by domain and research question, some recommend that, to use time series methods, a measurement requires at least 20 data points where possible (see Butner et al., 2015). However, with certain behaviors this will be unfeasible and the researcher will thus be forced to measure fewer data points at the expense of sensitivity. Furthermore, the question is how to specify a time unit at which the behavior should be quantified (e.g., per 30 s, 1 min, etc.). This value needs to be appropriate considering the behavior investigated and the situation in which it occurs. For example, measuring eating per 1-s intervals may be inappropriate in many contexts where people eat at a normal pace and do not take a new bite every second, in contrast to competitive eating, so a more sensible longer time interval would be required.

Once the appropriate behavior has been determined, one needs to examine how to construct a high-entropy situation in which this behavior will be investigated. Previous research suggested that such situations are characterized by uncertainty (Hirsh et al., 2012), internal conflicts (Vallacher et al., 1994; McKinstry et al., 2008; Duran et al., 2010, 2013), low-level interpretations (Vallacher and Wegner, 1987, 2014), or unfamiliarity with the behavior that needs to be performed (Stephen et al., 2009b). Hence, it is plausible that high-entropy situations are the ones where previous goals, habits, skills, or experiences cannot easily inform the person of an appropriate course of action. The best way to construct a high-entropy situation is probably by selecting an unfamiliar behavior without mentioning any purpose regarding why it is being performed, or by giving ambiguous instructions. Another option is to choose a familiar behavior (e.g., eating) while employing unfamiliar type of stimuli (e.g., foods with unusual shapes) and/or providing ambiguous or incoherent instructions (e.g., specifying that the foods are made of unknown exotic ingredients, or providing many different conflicting purposes regarding why the behavior needs to be performed). Alternatively, a high-entropy situation could be initiated by creating disorder within the environment where the behavior is being enacted (see Stephen et al., 2009b).

Selecting an appropriate prime is also essential. Researchers have devised many priming manipulations over the past 20 years and conceived different ways to classify them (e.g., Förster and Liberman, 2007; Loersch and Payne, 2011; Molden, 2014; Wentura and Rothermund, 2014). Concerning the timing of administration, a priming manipulation can be displayed either before the behavior of interest or simultaneously. For example, visual primes, such as colors, posters, etc. can be administered alongside a behavior by being embedded in the context of action (e.g., computer screen; Mandel and Johnson, 2002; Mehta and Zhu, 2009). Other priming manipulations, such as a scrambled sentences task (Shariff and Norenzayan, 2007), remembering an event from one's past (Lee and Schnall, 2014), or watching a video (Schreibman et al., 2000) can only be administered prior to a behavior. In general, a researcher can use either type of priming manipulations to study emergence, assuming they are theoretically linked to a behavior of interest. However, I discourage using subliminal primes because the effects of such primes may be short-lasting and incompatible with more complex behaviors captured as time series (Aarts et al., 2008).

Specifying how to capture changes in cognitive entropy is the final prerequisite for investigating behavioral priming under emergence. As previously suggested, this can be done by tracking participants' bodily movements and translating them into a motion time-series (e.g., change in velocity; Stephen et al., 2009b). For this purpose, it is possible to use professional motion tracking devices (e.g., Stephen et al., 2009a,b; Duran et al., 2013), track mouse movements on the computer screen (e.g., McKinstry et al., 2008; Freeman and Ambady, 2010), or video-record a participant throughout the experiment and extract the motion time-series using open-source software (e.g., Westlund et al., 2015) or commercial software (e.g., Matlab Computer Vision System Toolbox). These methods could be used in isolation, but combining them may also be interesting to see whether they would yield convergent results. Motion time-series are usually sampled at high frequencies (e.g., 100 times per second; Stephen et al., 2009b), which means they consist of many data points. In this context, information entropy can be computed using recurrence quantification analysis (RQA) that is suitable for such relatively large time series (for tutorials, see Pellecchia and Shockley, 2005; Webber and Zbilut, 2005; for statistical packages, see Coco and Dale, 2014; Garcia, 2015; Rawald et al., 2017). Computing other entropy measures, such as sample entropy, would also be plausible (Richman and Moorman, 2000).

Considering all the options reviewed above, priming under emergence could be investigated in numerous ways. Here I propose a prototypical research design that can be easily modified to accustom different possibilities—a 2 (*situation*: high vs. low entropy) × 2 (*priming manipulation*: 1 vs. 2) between-subjects design that yields four conditions: Condition 1 (high-entropy situation + prime 1), Condition 2 (high-entropy situation + prime 2), Condition 3 (low-entropy situation + prime 1), and Condition 4 (low-entropy situation + prime 2). The *situation* variable refers to whether the situations in which the behavior of interest, which should be the same for all participants, is introduced to them activate high vs. low cognitive entropy. The *priming manipulation* variable refers to the type of priming procedure used; primes 1 vs. 2 indicate any two priming manipulations that induce different mental constructs (e.g., Dijksterhuis and Van Knippenberg, 1998).

**Figure 6:**
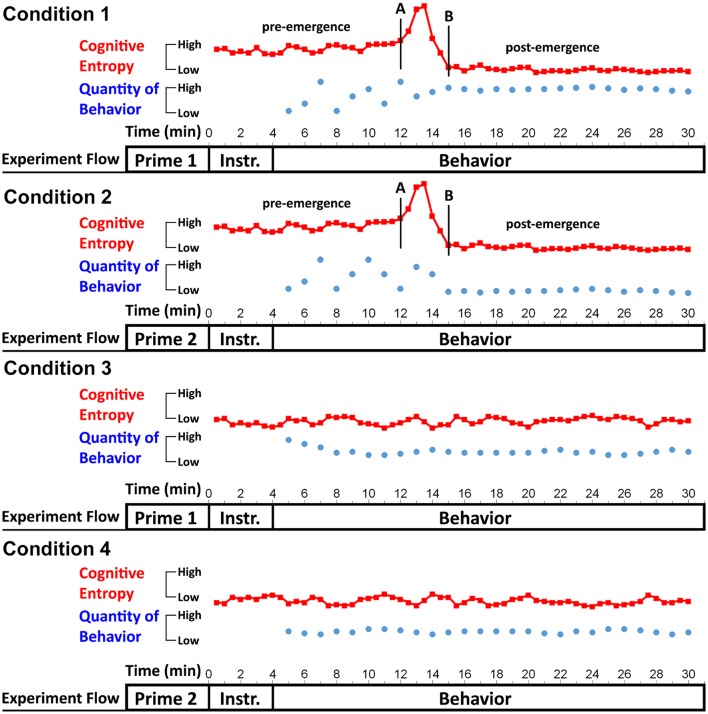
A schematic representation of a prototypical experiment investigating emergence. On one level, the figure depicts experimental flow, where a priming manipulation should be administered first, followed by experimental instructions (*Instr.)* introducing the behavior being investigated, and finally the behavior should be captured as a time series (in the present graph, the behavior is sampled per 1-min intervals). The figure also depicts idealized data patterns demonstrating that priming may influence behavior only in high-entropy situations (Conditions 1 and 2), where emergence is likely to take place, but not in low entropy situations (Conditions 3 and 4). In the former situations, primes evoking different cognitive constructs (Prime 1 vs. 2) may result in the emergence of distinct behavioral patterns (represented by blue dots) characterized by different fixed-point attractors, whereas this may not be the case in low-entropy situations. Fluctuations of cognitive entropy (per 30-s intervals) are depicted by the red squares.

Figure 6 constitutes a schematic representation of the proposed experiment. On one level, the figure depicts experimental flow. In each condition, participants should first receive a priming manipulation. Furthermore, they should be presented with experimental instructions (labeled as *Instr*.) conveying the behavior of interest in a way that induces either high or low cognitive entropy (depending on the condition to which they have been allocated), and then undertake the behavior (measured as a time series). In the figure, the quantity of behavior is computed per 1-min intervals (the duration should be appropriate in the context of the behavior as previously discussed). It is worth noting that participants will differ regarding the time taken to complete the behavioral task, but the researcher should ensure that the task is sufficiently long for even the quickest participants to yield an appropriate number of data points as previously discussed.

As can be seen from Figure 6, cognitive entropy (red line with the squares) should be assessed even while participants are reading experimental instructions, given that it is important to show that the instructions in the high-entropy condition indeed yield higher cognitive entropy relative to the low-entropy condition. The red squares also indicate that entropy scores in the figure are computed from time series of participants' bodily motion every 30 s. I suggest that entropy is always computed at shorter time intervals than the behavior itself (e.g., 2–3 entropy scores per each time unit at which the behavior is assessed) because this may provide more sensitive insights into the cognition behind the emergence of the behavioral pattern. Although it is not clearly specified how many data points RQA requires to reliably calculate entropy from a time series of bodily motion, 300 or more data points should be sufficient (for more comprehensive discussion, see Aks, 2011). Considering that motion tracking can yield numerous data points per second (Pellecchia and Shockley, 2005), in some cases even just a few seconds of data would be sufficient for entropy calculation.

Figure 6 also depicts idealized data patterns from which hypotheses that need to be met to demonstrate that priming influences behavior only under emergence can be extrapolated. As Hypothesis 1, I propose that priming will change behavior only in high-entropy situations (Conditions 1 vs. 2), but not under low entropy (Conditions 3 vs. 4). In this regard, to change behavior means to produce different attractors, given that, in high-entropy situations, the two priming manipulations may result in different prime-consistent behavioral patterns. To investigate this, one could utilize the tools of Butner et al. (2015) and compute attractors for each participant's behavioral time series by fitting Equation (1). Indeed, we are interested only in single fixed point attractors, given that I speculate the emergence is most likely to result in an attractor that is stable and does not further change, as in Figure 6. Then, one should use the attractor value for each participant as the dependent variable and compute an interaction between *situation* and *priming manipulation* (Aiken and West, 1991; Hayes, 2013). The interaction should be significant, given that only attractors in Conditions 1 and 2 (but not in Conditions 3 and 4) are expected to differ. Additionally, one could probe attractor strength as the dependent variable. From Graph 6, we can see that the behavior in Conditions 3 and 4 (vs. 1 and 2) stabilizes relatively more quickly, which means these conditions should yield stronger attractors (Butner et al., 2015). However, in some circumstances even Conditions 1 and 2 may differ in attractor strengths; for example, if a priming manipulation itself paces up the process of emergence.

To construct Hypothesis 2, we need to take a close look at the cognitive entropy lines in each of the four conditions in Figure 6. The lines indicate that, in Conditions 1 and 2 where emergence occurs, entropy may start at a relatively higher level than in Conditions 3 and 4, reach its peak between the points A and B that comprise the emergence region, and then drop to a lower level (Stephen et al., 2009b). The timing of this process may differ across participants, but I speculate it would follow a similar pattern. In contrast, in Conditions 3 and 4, entropy may remain relatively stable. I propose this difference could be quantified using two values—standard deviation of the distribution of entropy points computed for each participant as well as the maximum entropy point. Therefore, according to Hypothesis 2, distribution of entropy values for participants in the high (vs. low) situation entropy conditions should have relatively higher standard deviation, and these participants should also have higher maximum entropy.

Finally, it is essential to show that the *situation* variable indeed influenced cognitive entropy levels. In other words, while reading experimental instructions (which serve as the manipulation of the situation), participants in the high (vs. low) entropy conditions should experience elevated cognitive entropy (Hypothesis 3). To investigate this, the researcher could compute average cognitive entropy over the duration of experimental instructions for each participant and then conduct *t*-tests, ANOVAs, or regressions comparing this measure for Conditions 1 and 2 vs. Conditions 3 and 4. Overall, an experiment supporting all the three hypotheses would support the notion that priming affects behavior only under emergence.

The experimental design I proposed can be adapted to different priming manipulations and behaviors. For example, instead of priming people before the behavioral task, one can use a prime that can be presented alongside the behavior. The biggest challenge is in fact how to deal with behaviors that are inherently linked to high-entropy cognitive states because they are novel to the extent that they involve many possible interpretations. In that case, it would be difficult to experimentally create a low-entropy situation. Therefore, the researcher could use one set of experimental instructions for all participants, and then assess their cognitive entropy while reading the instructions. Given that not all people are the same, some participants would probably experience higher cognitive entropy than others even in this case, and we could divide the participants into two groups—low vs. high cognitive entropy—based on their experiences. This variable could then be used as a moderator to test Hypotheses 1 and also to probe Hypothesis 2, whereas Hypothesis 3 is inherent in the variable itself.

Another difficulty may arise if the behavior cannot be assessed as a time series (e.g., one-off choices), in which case computing attractors via differential equations would not be plausible. In that case, one could assume that the choice itself constitutes an attractor and test Hypothesis 1 by analyzing the choice as a dichotomous dependent variable by applying logistic regression, and Hypothesis 2 may be tested on changes in cognitive entropy between the onset of the choice option and eventually making the choice (see McKinstry et al., 2008). Overall, there are many creative ways to investigate priming in the context of emergence, and given that standard procedures have not yet been established I encourage researchers to probe different possibilities.

### Readjustment

Another type of influence concerns situations in which the impact introduced in Section Emergence is unlikely to occur because it is clear what the actor needs or wants to do and her/his goals (Moskowitz and Grant, 2009), habits (Aarts and Dijksterhuis, 2000), and/or personality traits (Vallacher et al., 2002a) are likely to quickly “take control” over the behavior. In other words, these are the situations that have an inherent strong attractor. For example, a person late for work is likely to walk quickly as soon as leaving the house, which means that quick walking will ensue from the start and comprise a strong attractor. Another example is an unrestrained eater who finds her/himself in a familiar eating situation. Because unrestrained eaters usually have one strong attractor for eating (e.g., Fedoroff et al., 1997), the person is likely to quickly settle on an eating pattern that corresponds to this attractor.

To remove any natural system from a strong attractor, a substantial external perturbation is necessary (Prigogine and Stengers, 1984; Vallacher et al., 2015). Based on this premise known to apply to various natural systems, ranging from an embryo to bacteria and even to cognitive systems (Prigogine and Stengers, 1984; Ward, 2002; Gallopín, 2006), it is plausible that priming may not considerably impact behaviors driven by strong attractors. In this context, I speculate that any priming effect, if it occurs at all, may be relatively minor and short lasting: it should not change the attractor value but only slightly decrease its strength, depending on whether the prime is compatible or incompatible with the attractor (Vallacher et al., 2002a).

As a hypothetical example, let us consider a person whose work starts in 30 min but who left the house too late and thus needs to hurry. This person's walking behavior will be driven by the goal to arrive to work as quickly as possible, and may therefore follow a stable pattern of distance over time corresponding to quick walking (e.g., roughly 50 meters per each 30-s interval of the 30-min period). If this person encounters a compatible prime that activates the mental construct of “walking quickly,” a behavioral impact is unlikely, given that the person is already walking quickly. An incompatible prime (walking slowly) may, in contrast, briefly decrease the speed of walking (e.g., over few 30-s intervals), but the goal to arrive to work as quickly as possible will soon take over and the fast pace of walking will resume. If we computed the attractor for the person's time series of the distance walked per 30-s intervals in situation A (compatible prime) vs. situation B (incompatible prime), we would probably see that the attractor value itself would stay relatively similar, and only the attractor strength may be smaller in situation B (Butner et al., 2015). This is the type of priming influence to which I refer as “readjustment,” given that the behavior of interest is briefly impacted by an incompatible prime but quickly readjusts by returning to its initial attractor state (see Vallacher et al., 2002a).

#### Investigating readjustment

When designing an experiment to demonstrate readjustment, three prerequisites need to be met. First the researcher needs to select a situation where a person's goals, habits, personality traits, and/or previous experiences can easily manifest themselves in the shape of strong behavioral attractors. These are essentially low-entropy situations as discussed in Section Investigating Emergence. Second, it is imperative that the population being tested has one strong behavioral attractor that should occur in that situation, rather than multiple possible attractors of different strength, given that under those circumstances priming may lead to attractor switch rather than readjustment (see Section Attractor Switch). This can be established by carefully researching relevant literature. For example, I already referred to restrained eaters as the type of population that may have two attractors—one for restrained and one for enhanced eating (Fedoroff et al., 1997)—and unrestrained eaters may therefore be more suitable for studying readjustment in relation to eating. Finally, the researcher needs to determine psychological variables that strongly predict the behavior being investigated. These can be any person variables, ranging from the Big Five Personality Traits (Costa and McCrae, 1992) to more specific constructs such as behavioral inhibition/activation system (Carver and White, 1994) or need for cognition (Cacioppo et al., 1996), implicit attitudes (Greenwald and Banaji, 1995), goals (Little, 1983), habits (Verplanken and Melkevik, 2008), and even demographic variables including gender, age, education, etc. (Teo, 2001). Relevant psychological variables should be measured in the experiment because they are likely to determine the value of the attractor (Vallacher et al., 2002a) and may therefore allow specifying whether priming manipulations used are compatible or incompatible with the attractor.

**Figure 7:**
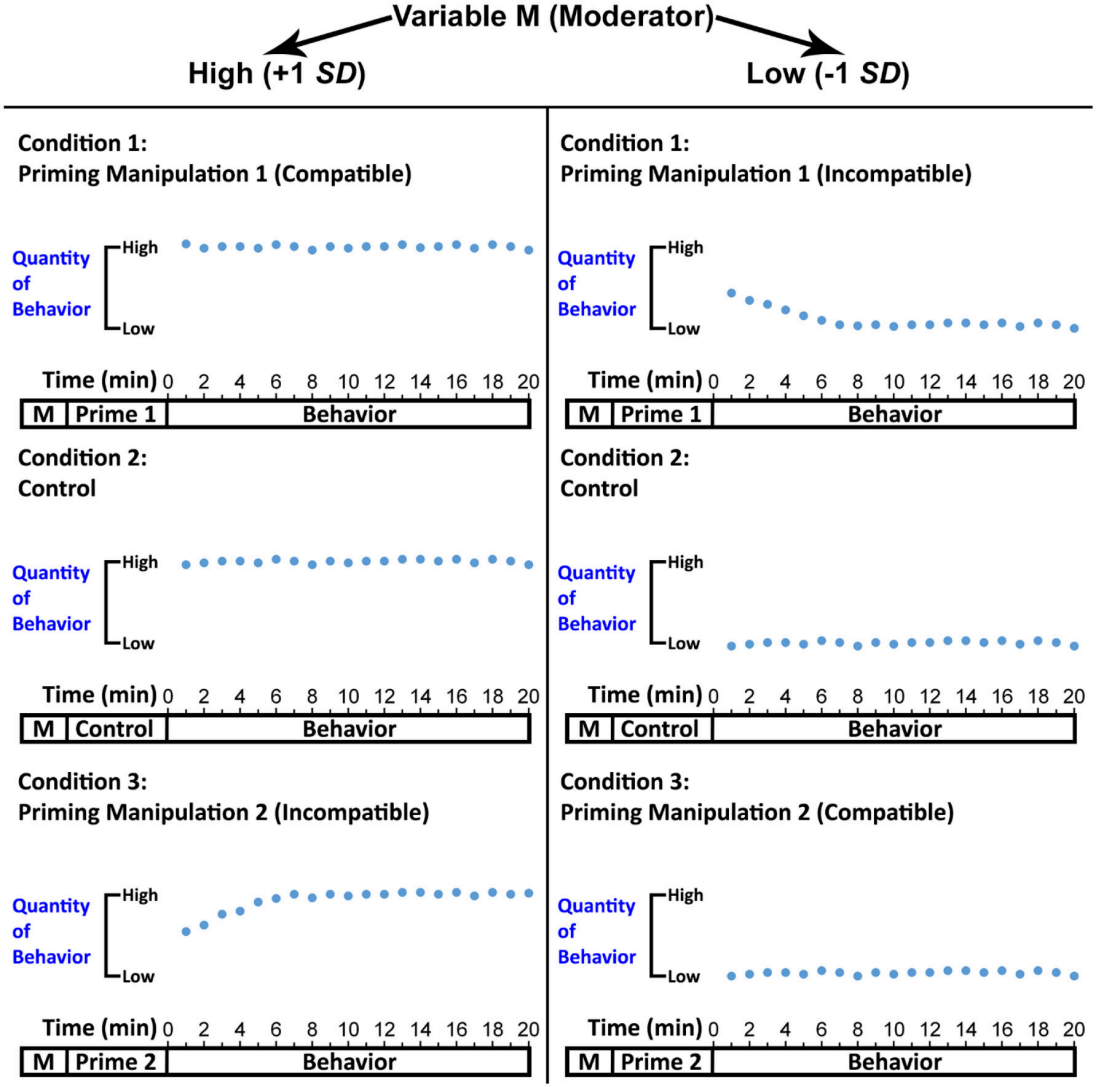
A schematic representation of a prototypical experiment investigating readjustment. On one level, the figure depicts experimental flow, where the Moderator Variable M should be assessed first, followed by the administration of the priming manipulation. Finally, the behavior of interest should be introduced (the instructions are not shown in the graph due to space constraints) and captured as a time series (in the present graph, the behavior is sampled per 1-min intervals). The figure also depicts idealized data patterns demonstrating that readjustment may occur when a cognitive construct activated by priming is incompatible with the expected attractor value (expected attractor value is determined by the moderator: at +1 *SD* a high quantity of behavior is expected, whereas at −1 *SD* a low quantity of behavior is expected). Hence, at high values of the moderator (+1 *SD*), readjustment may occur for the incompatible Prime 2 (vs. control), but not for the compatible Prime 1. The opposite would be the case at low values of the moderator (−1 *SD*).

Figure 7 constitutes a schematic representation of a prototypical experiment investigating readjustment. At the beginning of this experiment, it is crucial to assess a psychological variable (*Variable M*) that determines attractor dynamics as indicated above to be used as a continuous moderator of the impact of priming on attractor strength. Thereafter, participants should be subjected to the relevant priming manipulation. As depicted in Figure 7, *priming manipulation* could be a between-subjects variable consisting of three levels (Prime 1, Control, Prime 2). Prime 1 should be compatible with high values of Variable M. For example, if Variable M corresponds to a person's hunger, and high values of this variable indicate very hungry, then Prime 1 should encourage eating, which is compatible with being hungry. In contrast, Prime 2 should be compatible with low values of Variable M (= satiated), which means it should discourage eating. In the control condition, participants should complete a neutral task (e.g., the one that does not prime eating constructs). Finally, all participants should undertake a time-series compatible behavior of interest. In Figure 7, I do not refer to instructions introducing the behavior given space constraints, but I assume the instructions are part of the experimental flow.

To produce the dependent variable, one would need to fit Equation (1) to each participant's behavioral time series and compute attractor strength (Butner et al., 2015). An analysis of simple slopes (Aiken and West, 1991; Hayes, 2013) could then be performed, with Variable M as a continuous moderator, priming manipulation as a categorical independent variable (it would comprise two dummy variables, one for Prime 1 and one for Prime 2), and attractor strength as the dependent variable. This analysis would compute the impact of Primes 1 and 2 vs. the control condition on attractor strength at values of the moderator compatible or incompatible with the primes.

The overarching prediction (Hypothesis 1) would be that the impact of priming on attractor strength will change at different values of the moderator, thus yielding a significant interaction effect. At high values (+1 *SD*), I speculate that the incompatible Prime 2 (vs. Control) would decrease attractor strength, whereas Prime 1 would have no influence, given that it encourages the behavior in the same direction that is already facilitated by Variable M (Hypothesis 2). In contrast, at low values (−1 *SD*) of M, the opposite may be the case (Hypothesis 3). In Figure 7, these predictions are reflected in the pattern of behavior. When Prime 2 is paired with high M, the first few data points have a tendency toward low quantity of the behavior, thus reflecting the short-lasting effect of the prime, but the behavior then stabilizes at high quantities, as would be expected given that high M should predict high quantity of the behavior. For Prime 1 and the control condition, the pattern of behavior is relatively stable because it starts at high levels and finishes at high levels. Hence, whereas under Prime 1 and the control condition attractor strength is expected to be similarly high, under Prime 2 it may be slightly lower given the readjustment has taken place. At low values of M, I speculate this would switch, and readjustment may now be found only for the incompatible Prime 1. To show that only readjustment took place in the experiment and there was no change of attractor values, one could compute these values (Butner et al., 2015) and show that no differences were found between Primes 1 or 2 and the control condition.

Although this experimental design is flexible and can be adjusted to different priming manipulations and behaviors, it is worth discussing whether readjustment should be investigated with time-series incompatible behaviors. In this regard, it would be possible to focus on behaviors that comprise numerous choices, such as online grocery shopping, and investigate whether priming influences the shopping behavior (e.g., the type of foods purchased) only very early in the process but not later, which would correspond to readjustment. My view is, however, that such behavioral measures are not sensitive enough to capture readjustment for several reasons. First, some participants may wait for too long to make the first choice for readjustment to be captured. Second, choice itself corresponds to an attractor that cannot be easily decomposed into different temporal units, and given that readjustment may not change the attractor but only its strength, capturing this type of influence may be difficult because it is unclear how to measure attractor strength with one-off choices (e.g., does the time needed to make a choice indicate attractor strength, or is it some other feature). Overall, when investigating readjustment, I recommend that researchers use time-series compatible behaviors.

### Attractor switch

So far, I discussed how priming can contribute to the emergence of a new behavioral attractor or slightly perturb an existing attractor that quickly readjusts. Another type of influence—attractor switch—can occur for individuals who have two or more attractors for one behavior, and one of these attractors is relatively weak, which means that relevant external perturbations can easily cause the other attractor to become dominant. An ideal example is the behavior of restrained eaters to which I keep returning in this paper. Restrained eaters have two fixed-point attractors for food consumption (Figure 1)—one of them corresponds to restrained eating (Attractor 1), and one to enhanced eating (Attractor 2). Hence, if these people are not exposed to any food primes (Fedoroff et al., 1997) or do not feel anxious (Heatherton et al., 1991), their eating pattern is likely to correspond to Attractor 1. However, if they are primed with food smell or feel anxious, the food intake is likely to correspond to Attractor 2 (Heatherton et al., 1991; Fedoroff et al., 1997).

**Figure 8:**
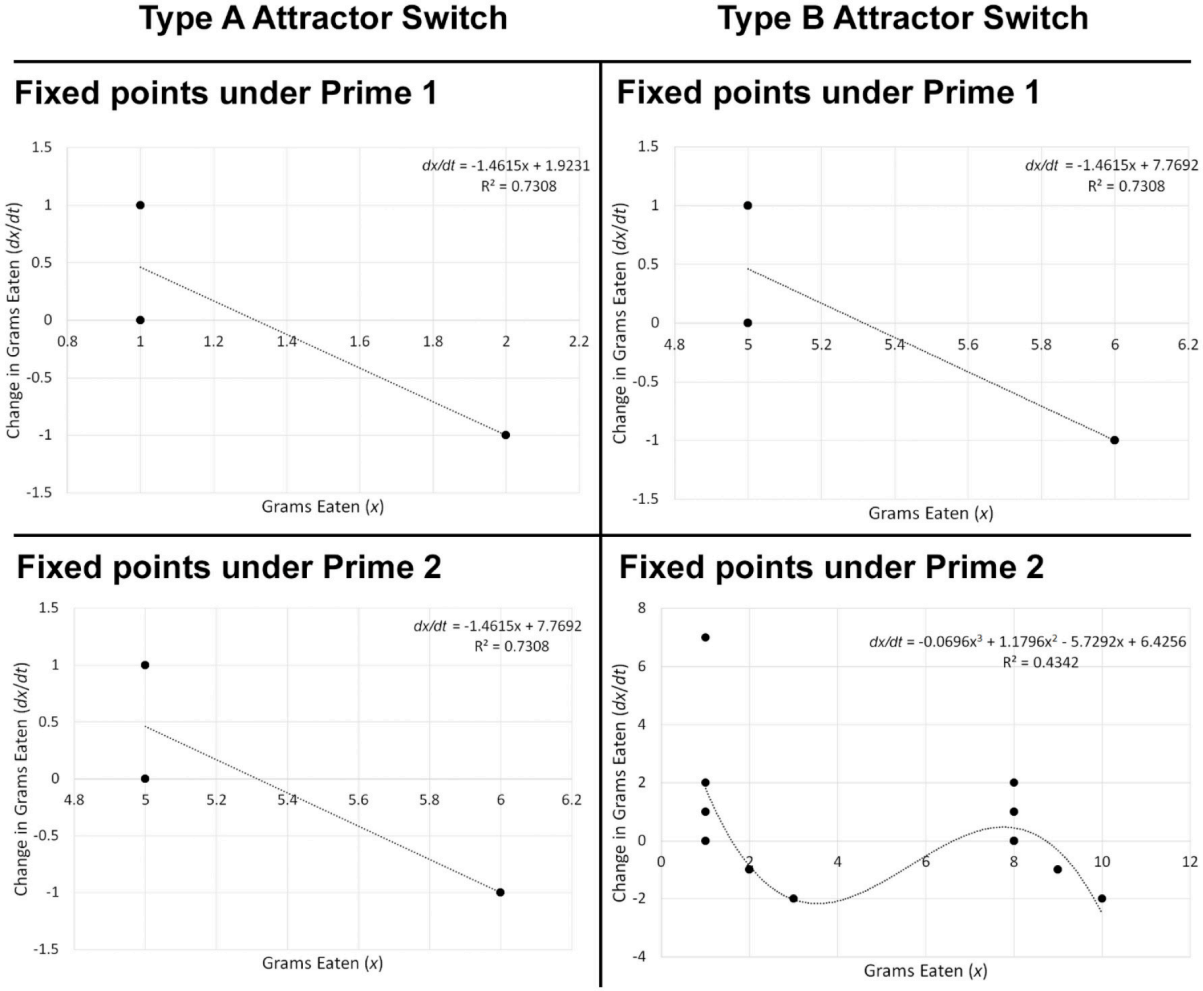
Examples of Type A and Type B attractor switch based on topologies of hypothetical food consumption data. When an attractor switch corresponds to Type A, some two priming manipulations should give rise to different attractors. In the example above, the fixed-point attractor present in the topology under Prime 1 is 1.316, whereas it corresponds to 5.316 under Prime 2. In contrast, when an attractor switch corresponds to Type B, some two priming manipulations should change the number of attractors. For example, under Prime 1, there is one fixed point attractor corresponding to 5.316. In contrast, under Prime 2, there are three fixed points: two attractors (1.597 and 8.733), and one repeller (6.618).

The difference between attractor switch and emergence is that, whereas under emergence a new behavioral attractor is being instigated under the influence of priming, attractors involved in attractor switch are already present in latent form and priming simply determines which one will become active (Coleman et al., 2007; Dixon et al., 2010). The example with restrained eaters corresponds to the simplest type of attractor switch, where only one attractor is likely to become activated in a given situation, depending on the priming manipulation previously encountered; I refer to it as Type A attractor switch (Figure 8). Although Type A has not been directly investigated in relation to priming by employing state of the art attractor computation techniques (Butner et al., 2015), research suggests that it may be a common behavioral priming effect (e.g., Fedoroff et al., 1997; Cesario et al., 2006).

There are other more complex types of attractor switch, to which I jointly refer as Type B. In a nutshell, this type of influence would occur if a priming manipulation not only changed the attractor itself, but instead changed the number of attractors, thus completely altering the system's dynamics. For example, if some Prime 1 led people to exhibit restrained eating, but another Prime 2 led them to exhibit both enhanced and restrained eating in the same situation, that would correspond to Type B attractor switch (Figure 8). In other words, Prime 2 would change the type of differential equation that best describes the behavioral change over time from Equations (1) to (3). In theory, Type B attractor switch can refer to a change of any simpler differential equation into any more complex differential equation, or the other way around. More precisely, it can refer to a system with n attractors transforming into one with n+1 (or n−1) attractors.

In the next section, I describe how to design research that would demonstrate attractor switch, with primary focus on Type A. The reason for this is two-fold. First, Type B attractor switch can be investigated using a variety of statistical models, and going into depth in this regard would require a separate article. Second, even if this type is methodologically possible, research so far does not indicate to what extent its occurrence in relation to behavioral priming may be plausible. Hence, Type A attractor switch may be more relevant given the current state of knowledge.

#### Investigating attractor switch

The first step in probing Type A attractor switch is identifying individuals who may have two attractors in relation to a behavior being investigated. One possibility is to first consider different personality characteristics that may suggest multiple attractors. Beyond restrained eating that I already discussed, one example is self-concept clarity, which concerns the extent to which people think they know themselves and their self-concepts are stable (Campbell et al., 1996). People with low self-concept clarity usually have unstable perceptions (= multiple attractor states) of their own ability to succeed in different situations and their behavior may therefore be susceptible to primes encountered in these situations (Campbell, 1990). Another option to identify people characterized by multiple attractors is to consider dissociation between implicit and explicit measures of certain cognitive constructs. Indeed, people's attitudes and self-views may have two components—implicit and explicit—that can be either congruent or discrepant (Hofmann et al., 2005). For example, a person may have negative explicit but positive implicit attitudes regarding candies (Hofmann et al., 2007, 2008), or high explicit but low implicit self-esteem (Asendorpf et al., 2002). Research suggests that explicit attitudes or self-views rely on self-control, and given that self-control is fragile, they can be disrupted by relevant external forces that will cause implicit attitudes and self-views to take over (Strack and Deutsch, 2004; Vohs, 2006; Hofmann et al., 2009). Hence, priming may impair the dominance of explicit forces in shaping behavior, thus causing the behavioral attractor to switch.

Finally, one possibility is that individuals with multiple attractors can be determined if we assess their cognitive entropy (via motion tracking) while they are answering a personality questionnaire or some other behaviorally relevant measure. Indeed, given that higher cognitive entropy indicates uncertainty (Hirsh et al., 2012), it may uncover individuals who are less certain of how they view themselves regarding the construct being assessed and therefore have multiple attractors. Overall, to capture Type A attractor switch, individuals with multiple attractors need to be clearly specified. Otherwise, any priming manipulations are likely to result only in readjustment, as discussed in Section Readjustment.

Determining an effective priming procedure is also important, given that it needs to be directed at the weaker rather than the stronger behavioral attractor, or else the switch is unlikely to occur (Vallacher et al., 2015). For example, we know that, for restrained eaters, restrained eating is a weaker attractor that may be disrupted by relevant perturbations, and once the stronger attractor (enhanced eating) has been activated, it may be difficult to switch it back to restrained eating (Heatherton et al., 1991). In general, it is plausible that attractors determined by explicit attitudes or self-concepts are always weaker, given that self-regulatory forces on which they rely are fragile, and external perturbations can therefore influence attractors consistent with implicit attitudes or self-concepts to occur (Hofmann et al., 2009). Hence, designing an appropriate experiment would require one control priming manipulation that is either neutral or evokes a cognitive construct that encourages behavior consistent with the weaker attractor and will not cause any change (e.g., Prime 1 in Figure 8-Type A Attractor Switch which is consistent with restrained eating), and one manipulation that is inconsistent with the weaker attractor and can thus disrupt it to enforce the other attractor (e.g., Prime 2 in Figure 8-Type A Attractor Switch which fosters enhanced eating). Therefore, the experimental design would involve a between-subjects variable—*priming manipulation* (Prime 1 vs. 2).

Behavior itself should ideally be measured as a time series, and the hypothesis is that Prime 1 will on average lead to a different attractor compared to Prime 2 (Figure 8 depicts two hypothetical topologies that are in line with this prediction). Although time-series compatible behaviors are desirable, I suggest that the incompatible behaviors may also be sensitive enough to capture Type A attractor switch. For example, if the dependent variable is a one-off choice, we can treat it as a dichotomous outcome and analyze the impact of priming using logistic regression. Showing that a priming manipulation increased the odds of one behavior over another would indicate that it created Type A attractor switch, given that the two choices themselves correspond to two different behavioral attractors.

Attempting to probe Type B attractor switch could complicate matters because, based on previous research, it may be difficult to determine when exactly a priming manipulation would create this kind of impact. On a methodological level, modeling may also get difficult depending on the complexity of change taking place. The simplest way to test Type B attractor switch would involve comparing the number of attractors under Prime 1 vs. Prime 2. For example, the researcher could attempt to fit polynomial regression models of different order to each participant's behavioral time series, identify the one with the best fit, and count the number of fixed point attractors (Figure 8). This number could be used as a categorical dependent variable, and the impact of priming on this variable investigated via ordered logistic regression. A significant influence would indicate that Type B attractor switch has taken place.

Another possible method of investigation involves catastrophe modeling, which is frequently used by the DSP researchers (e.g., Zeeman, 1977; Latané and Nowak, 1994; Guastello, 1995; Liu and Latané, 1998; Guastello et al., 2012) to investigate change in the number of attractors as a function of a splitting factor (in our case, priming would be the splitting factor). Resources that can be consulted for catastrophe modeling include Cobb (1981), Grasman et al. (2009); Guastello (2011a,b); Van der Maas et al. (2003), and Zeeman (1976).

## Discussion

In the present article, I argued that the DSP offers conceptual and methodological tools that can improve the understanding of when exactly behavioral priming effects are likely to occur and allow precise measurement of these effects. Three main types of the impact of priming on human behavior were identified: Emergence, Readjustment, and Attractor Switch. Emergence may be expected to occur in situations that are likely to involve high cognitive entropy. In these situations, the actor's cognition is marked by different possible perceptions or interpretations, and a clear course of action is therefore missing (Hirsh et al., 2012). Given the absence of a strong attractor, the actor is likely to be sensitive to external perturbations such as primes, which may inform the emergence of a new stable behavioral attractor, thus lowering cognitive entropy in the process and allowing cognition to remain functional in meeting situational demands (Stephen et al., 2009b; Dixon et al., 2010; Vallacher et al., 2010).

However, not all situations are marked by high cognitive entropy, given that they involve demands and outcomes the actor is familiar with, and her/his goals, habits, personality traits, and previous experiences are likely to manifest themselves in well-established attractors (Vallacher et al., 2002a). I argue that the underlying attractor structure may then determine the impact of priming on behavior. If one strong behavioral attractor is dominant, priming may have a minor impact on behavior and influence only attractor strength but not its value—a type of influence to which I refer as readjustment (Butner et al., 2015). If, on the contrary, the person has two potential attractors, one of which is relatively weaker, priming may disrupt the occurrence of the weaker attractor and result in the onset of the stronger one—a type of influence to which I refer as attractor switch (Type A). More complex types of attractor switch are also possible (Type B) and can comprise a change in the number of attractors manifested in a situation (Zeeman, 1977; Guastello, 2011a,b; Butner et al., 2015). However, based on previous research regarding behavioral priming, it is unclear whether and when exactly such complex influences should occur.

To demonstrate how to investigate the three types of effects, I introduced some of the relevant tools of dynamical modeling that have not been implemented by priming researchers so far and showed how to combine them with more traditional methods. For example, I demonstrated how Butner et al. 's (2015) approach of computing attractors and their strength by employing differential equations, as well as the computation of information entropy based on behavioral time series (Shannon, 1948; Stephen et al., 2009b; Guastello, 2011c), can be embedded into traditional research designs (e.g., between-subjects) to probe emergence, readjustment, or attractor switch. Overall, the present article demonstrated that concepts and methodological tools used by the DSP researchers can enrich the science of behavioral priming.

### Limitations

Although drawing on insights from the DSP allows profound understanding regarding potential priming influences on behavior and how they should be investigated in different circumstances, there are certain limitations to using methodology stemming from the DSP when studying behavioral priming. First, many behaviors in which priming researchers are interested, such as eating or solving intellectual tasks, can be computed only as relatively short time series, which may be sufficient for identifying simple attractor structures, but may prevent more complex dynamical modeling that could provide insights into intricate attractor dynamics (Guastello and Gregson, 2011). Some behaviors are in fact time-series incompatible, which means that dynamical modeling of attractor strength is difficult to achieve, and sensitive types of influence such as readjustment may be hard to capture (Butner et al., 2015).

Furthermore, the type of data that behavioral priming can yield is in most cases not suitable for being modeled as a multidimensional dynamical system. For example, if we wanted to model how a specific behavior (e.g., eating) changes alongside another relevant variable (e.g., hunger) over time, which would comprise a two-dimensional system (Butner et al., 2015), we would need to measure both eating and hunger at identical temporal intervals (e.g., every minute). This is easier said than done because asking participants how hungry they feel every minute is intrusive and could destroy the behavioral dynamics that would otherwise evolve. Hence, my suggestion is to use only variables that can be measured without self-reports (e.g., physiological measures) alongside behavior to model multidimensional dynamical systems, but such measures may not always provide us with desired psychological information.

Despite these limitations, the DSP has profound implications for capturing behavioral priming effects and their replications, which is what I discuss next.

### Implications for priming effects and replications

If a priming researcher does not clearly understand dynamical principles on which priming is based, successfully capturing a priming effect of interest may be sheer luck. In this paper, I suggest that priming should be most likely to impact behavior only in situations where strong attractors are absent and need to emerge given high cognitive entropy. Based on this assumption, a priming researcher may unknowingly construct an unclear experimental situation, or simply test the type of participants who perceive the situation as such, and obtain strong priming effects on behavior (e.g., the mean value of behavior under one priming manipulation being different than under another manipulation). However, if a conceptual or direct replication of this study is undertaken, the situation or type of participants may slightly change, thus changing the effect type to readjustment or attractor switch, which may cause the replication to fail because the researcher did not consider the factors and statistical tools necessary to capture either of these effects.

In reality, it is quite possible that, in a single priming experiment, emergence, readjustment, and attractor switch may occur for different participants. Some participants may find the experimental situation ambiguous and thus experience emergence, whereas some participants may find the situation clear and either experience readjustment or attractor switch, depending on the underlying attractor structure. If the experiment has not been designed to probe such a complex dynamic, it can easily fail to capture any behavioral priming effects, or capture them by chance. It is only possible to guess how many priming experiments have failed because the design and data analysis have not been approached from an accurate methodological perspective.

Finally, these insights allow me to discuss the role of replications in “saving the field” and determining which priming effects are robust. Given that, from the perspective of dynamical systems, priming researchers so far have not been designing their research in a way that would accurately capture priming effects, attempting to replicate the research does not necessarily make sense because the replications do not lead to improved understanding of behavioral priming. In fact, they may lead to the conclusion that the impact of some priming manipulations on behavior is not robust, whereas in reality it may be robust if measured in an appropriate manner. Even successful replications may not be informative because they may not provide an insight into why a robust priming effect occurs (e.g., because the situation evokes high cognitive entropy). Therefore, my position is that priming effects should first be investigated by employing dynamical modeling, and only once a more profound understanding of these effects has been achieved as a result replications should ensue. Otherwise, replication efforts may be in vain because of repeating the same old mistakes that hampered the understanding of behavioral priming in the first place.

## Author contributions

DK is the sole author of the present manuscript: he conceived the manuscript, wrote it, and prepared it for submission.

### Conflict of interest statement

The author declares that the research was conducted in the absence of any commercial or financial relationships that could be construed as a potential conflict of interest.

## Appendix

### Recommended reading

#### Theoretical resources

Bar-Yam, Y. (1997). *Dynamics of Complex Systems*. Reading, MA: Addison-Wesley.
Beer, R. D. (2000). Dynamical approaches to cognitive science. *Trends Cogn. Sci*. 4, 91–99. doi: 10.1016/S1364-6613(99)01440-0
Gros, C. (2008). *Complex and Adaptive Dynamical Systems: A Primer*. New York, NY: Springer.
Guastello, S. J., Koopmans, M., and Pincus, D. (2009). *Chaos and Complexity in Psychology: The Theory of Nonlinear Dynamical Systems*. New York, NY: Cambridge University Press.
Spivey, M. J. (2007). *The Continuity of Mind*. New York, NY: Oxford University Press.
Vallacher, R. R., and Nowak, A. (1997). The emergence of dynamical social psychology. *Psychol. Inq*. 8, 73–99. doi: 10.1207/s15327965pli0802_1
Ward, L. M. (2002). *Dynamical Cognitive Science*. Cambridge, MA: MIT press.

#### Methods and data analysis

Butner, J. E., Gagnon, K. T., Geuss, M. N., Lessard, D. A., and Story, T. N. (2015). Utilizing topology to generate and test theories of change. *Psychol. Methods* 20, 1–25. doi: 10.1037/a0037802
Guastello, S. J., and Gregson, R. A. M. (eds.) (2011). *Nonlinear Dynamical Systems Analysis for the Behavioral Sciences Using Real Data*. Boca Raton, FL: CRC Press; Taylor and Francis.
Holden, J. G., Riley, M. A., Gao, J., and Torre, K. (2013). Fractal analyses: Statistical and methodological innovations and best practices. *Front. Physiol*. 4:97. doi: 10.3389/fphys.2013.00097
Riley, M. A., and Van Orden, G. C. (2005). *Tutorials in Contemporary Nonlinear Methods for the Behavioral Sciences*. Available online at: http://www.nsf.gov/sbe/bcs/pac/nmbs/nmbs.jsp (Accessed March 1, 2005).
